# Supplementary material for: Microbial Succession under Freeze–Thaw Events and Its Potential for Hydrocarbon Degradation in Nutrient-Amended Antarctic Soil
Source: Microorganisms. 2021 Mar 16;9(3):609. doi: 10.3390/microorganisms9030609 (PMC8000410; doi:10.3390/microorganisms9030609)
Supplement: Supplementary file 1 [file microorganisms-09-00609-s001.pdf]

Table S1. List of 37 polycyclic aromatic hydrocarbon (PAHs) identified and quantified.

naphthalene (N)
C1-naphthalenes (C1N)
C2-naphthalenes (C2N)
C3-naphthalenes (C3N)
C4-naphthalenes (C4N)
acenaphthylene (ACF)
acenaphthene (ACE)
fluorine (F)
C1-fluorines (C1F)
C2-fluorines (C2F)
C3-fluorines (C3F)
dibenzothiophene (DBT)
C1-dibenzothiophenes (C1DBT)
C2-dibenzothiophenes (C2DBT)
C3-dibenzothiophenes (C3DBT)
phenanthrene (Ph)
C1-phenanthrenes (C1Ph)
C2-phenanthrenes (C2Ph)
C3-phenanthrenes (C3Ph)
C4-phenanthrenes (C4Ph)
anthracene (A)
fluoranthene (Fl)
pyrene (Py)
C1-pyrenes (C1Py)
C2-pyrenes (C2Py)
benzo[*a*]anthracene (BaA)
chrysene (Ch)
C1-chrysenes (C1Ch)
C2-chrysenes (C2Ch)
benzo[*b*]fluoranthene (BbFl)
benzo[*k*]fluoranthene (BkFl)
benzo[*e*]pyrene (BePy)
benzo[*a*]pyrene (BaPy)
perylene (Per)
indeno [*1,2,3-cd*]pyrene (I-Py)
dibenzo[*a,h*]anthracene (DBahA)
benzo[*ghi*]perylene (BghiPer)
